# Characteristics of Patients Discontinuing Care

**DOI:** 10.3390/dj7020031

**Published:** 2019-03-28

**Authors:** Lisa Simon, Gurmukh Singh Dhaliwal, Chieh-Han Jeffrey Liu, Pranshu Sharma, Shernel Thomas, Sarah Bettag, Katherine G. Weber, Peggy Timothé, Romesh P. Nalliah

**Affiliations:** 1 Oral Health and Medicine Integration, Harvard School of Dental Medicine, Boston, MA 02115, USA; Lisa_Simon@hms.harvard.edu; 2 Harvard Medical School, Boston, MA 02115, USA; 3 School of Dentistry, University of Michigan, Ann Arbor, MI 48109, USA; gurridhaliwal@gmail.com (G.S.D.); jeffreyliu80330@gmail.com (C.-H.J.L.); pranshu.sh@gmail.com (P.S.); shernel@umich.edu (S.T.); sbettag@umich.edu (S.B.); kgweber@umich.edu (K.G.W.); 4 Dental Public Health Residency, Texas A & M School of Dentistry, Dallas, TX 75246, USA; ptimothe@tamhsc.edu

**Keywords:** oral health care access, access disparities, cultural diversity/cultural competency, oral health education, limited English proficiency

## Abstract

**Objectives:** Due to lower fees, dental school clinics (DSCs) may provide dental care for vulnerable populations. This study evaluates factors associated with patients deciding to discontinue care at a DSC. **Methods:** This is a retrospective analysis of a patient transfer form that was implemented to smooth transition of a patient when their student provider graduated. Forms provided deidentified information about characteristics and unmet dental needs. Descriptive and bivariate statistics were used to identify associations between patient characteristics and deciding to continue treatment in the student practice. **Results:** Of 1894 patients, 73.4% continued care. Financial limitations were most commonly reported as the reason for discontinuing care (30.1%). Patients speaking a language other than English or who had reported financial barriers were significantly less likely to continue care. **Conclusions:** Dental school patients from vulnerable groups are more likely to discontinue care. Dental schools should implement programs that will assist patients in maintaining a dental home.

## 1. Introduction

Poor oral health is more prevalent in many vulnerable populations, including rural dwellers, people with low income, and people with limited English proficiency (LEP) [1]. These patients are also more likely to report that they are unable to access needed dental care, and as a result, are more likely to seek palliation for dental pain in hospital emergency departments, where there is often no definitive dental treatment available [2].

Increasing the number of dentists in the United States (US) who accept the government-funded insurance (Medicaid) and the number of dental services covered by Medicaid have been listed as measures to improve access to dental care for underserved groups [3]. Additionally, federally qualified community health centers must offer oral health services to the medically underserved areas in which they operate, although the scope of these services varies [4]. An alternative source of dental care for these patients may be dental school clinics (DSCs), where student dentists from one of the accredited US dental schools perform dental procedures under the supervision of licensed dentists [5].

The Commission on Dental Accreditation (CODA), which can be described as a continuous quality improvement organization for dental education, now requires all dental schools to meet competencies pertaining to cultural competence, ensuring that dentists graduating from these institutions are capable of providing effective and compassionate care to individuals from diverse backgrounds [6]. To do so, dental schools must cultivate diverse patient populations within their teaching clinics. Further, dental students who are exposed to vulnerable populations while training are more likely to treat these groups in future practice [7].

DSCs most often accept public insurance and have lower fee schedules than private dental offices. Although DSCs may provide more affordable care than private practices, dental appointments are often longer since procedures must be checked by licensed faculty. Additionally, it remains unknown whether the financial limitations and language barriers that put individuals at risk of poor oral health impact these individuals’ care in a dental school teaching practice. This may also impact the ability of dental schools to effectively train their students in the provision of culturally competent care. Moreover, DSCs face the additional challenge of a continuous cycle of providers due to graduation. It is not known how these transitions affect returning patients, recall, and compliance.

Previous studies on patients seeking care in DSCs have focused on the prevalence of specific medical conditions or have been adapted from consumer surveys used in other settings that do not address issues of quality and provider interaction [8,9,10,11,12]. The current pilot study is a retrospective analysis of the case disposition form at the Harvard School of Dental Medicine. This form was developed to communicate information to the new student provider that may not be readily available in the medical record. Care providers and patients share a unique relationship and patients may entrust their providers with information that may not be captured in medical history forms and other forms. For example, the patient may face transportation challenges because they have been banned from driving due to traffic infringements. When these important pieces of information are not communicated to the new student provider, there can be a conflict because the new provider may not know the personal barriers that may make it challenging for the patient to obtain dental treatment. Patients can feel embarrassed when required to regularly repeat the same information about their barriers to care whenever they have a new provider—a major challenge of receiving care in a school setting.

The case disposition form includes the chart number which enables the new provider to gain valuable information that the original student provider has learned while managing the patient. The case disposition form was designed to empower the new student provider to be a more understanding provider who can offer patient-centered care while reducing the need for relearning and rebuilding rapport. The purpose of the current study was to assess whether the barriers to care that patients faced were correlated to patients’ decisions to continue as a patient in the DSC after the graduation of their student provider.

## 2. Methods

**Figure 1:**
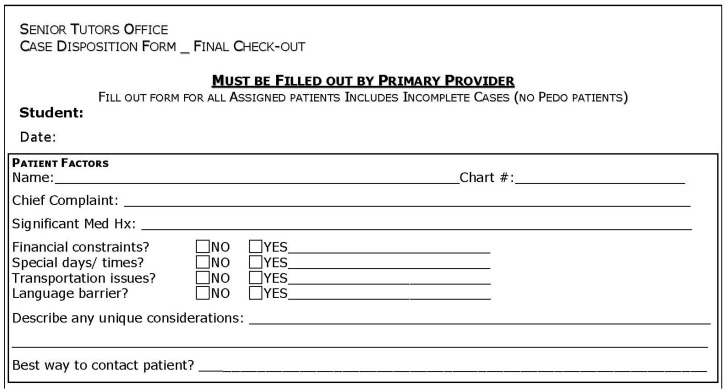
Sample patient disposition form.

All clinics at the Harvard School of Dental Medicine use AxiUm, an Exan product, as the electronic health record. In an effort to include information not present within the AxiUm interface, all students from the graduating class at Harvard also completed a paper patient disposition form for each patient they had seen in the school’s adult comprehensive care clinic (Figure 1). This form was designed to ensure a smooth transition of provider responsibilities from the graduating student to the junior classmate and communicate useful information that may not be in the AxiUm record. Only patients treated in the comprehensive care clinic and assigned to the student for care were considered—other limited care rotations, such as the oral surgery clinic, were excluded from paper disposition forms. These forms are used to assign patients with continuing needs to the appropriate student, advanced graduate education (resident), or faculty providers, as well as to evaluate students’ progression through the Doctor of Dental Medicine (DMD) curriculum. The disposition forms document a patient’s presenting chief complaint, medical history, the presence of financial constraints, time constraints, transportation constraints, lack of English proficiency, treatment required and rendered, and planned case disposition. Multiple constraints could be documented for each patient. Designated faculty advisors at the Harvard School of Dental Medicine, known as ‘Senior Tutors’, are responsible for the evaluation of these forms during ‘check-out’ meetings with graduating students. At the time of design and implementation of the patient disposition form, the second and third authors were serving as Senior Tutors at the Harvard School of Dental Medicine.

For internal purposes, patients’ chief complaints, medical history, presence or absence of barriers to care, and planned disposition were compiled in a spreadsheet. Preferred language was recorded if students indicated that the patient’s primary language was not English. Patient age and gender were not included. As the intent of these forms was to provide additional information for the reassignment of patients to new providers, the presence or absence of barriers was recorded as a binary variable; severity of barriers was not assessed. Patients who had no active dental disease and were on a recall schedule for routine dental exams, as well as patients with remaining planned dental treatments, were considered to be continuing care at the dental school. Patients who had reported that they did not wish to continue care, or patients who were lost to follow-up, were considered to be not continuing care at the dental school. The data were collected by a dental student and subsequently deidentified. The deidentified data from the classes of 2012 and 2013 were analyzed for this study. The study protocol was reviewed and approved as ‘not human subjects research’ by the Harvard School of Dental Medicine Institutional Review Board (IRB15-0341).

Descriptive and bivariate statistics were computed. *p* values were computed using a two-tailed *t*-test assuming equal variances to assess significant prevalence of barriers between patients electing to continue care and patients who did not continue care in the teaching practice (*p* ≤ 0.05 significance). Only predictor variables found to be significant in bivariate analysis were included in logistic regression. A multivariate logistic regression 4 model was used to identify associations and odds ratios of predictor variables with choice to continue care (*p* ≤ 0.05 significance). Age and gender were not included in the regression as they were not recorded on the disposition forms.

## 3. Results

A total of 1894 disposition forms were completed by the classes of 2012 and 2013 at the Harvard School of Dental Medicine. Of these, 1390 patients (73.4%) elected to continue care at the School (Table 1). Financial limitations were the most prevalent patient barrier to care reported by students, faced by 570 patients (30.1%). Time limitations were the next most common, experienced by 290 patients (15.3%). Students reported that 151 patients (7.97%) possessed limited English proficiency. Transportation barriers were faced by 118 patients (6.23%).

Of those patients with student-reported limited English proficiency, the most common preferred language was Spanish, spoken by 65 patients (43.05% of non-English-speaking patients, 3.43% of all patients) (Table 2). The second most common language was Portuguese (22.52% of non-English-speaking patients, 1.80% of all patients), followed by Russian (3.31% of non-English-speaking patients, 0.26% of all patients). Mandarin Chinese and Haitian Kreyol both had four speakers (2.65% of non-English-speaking patients, 0.21% of all patients).

When assessed with bivariate analysis, the group with limited time availability was not significantly less likely to continue care (*p* = 0.850) (Table 3). Groups with financial barriers, transportation limitations, or limited English proficiency were significantly less likely to continue care (*p* < 0.001, *p* = 0.007, and *p* < 0.001, respectively). 

In a logistic regression model, patients with either linguistic or financial barriers were significantly less likely to continue care (Odds Ratio (OR) 0.545, *p* = 0.001; OR 0.454, *p* < 0.001, respectively). The presence of transportation barriers was not found to be a significant predictor of discontinuing care (OR 0.737, *p* = 0.135).

Table 4 shows the distribution of constraints by chief complaint. Numerically, the largest number of constraints were financial and were associated with patients needing a crown, in pain, or needing a denture among those reporting a chief complaint. 

## 4. Discussion

Americans who have low incomes, speak a language other than English, or who must travel farther distances to reach a dentist are all at higher risk of reduced access to dental care [13,14,15,16]. These populations are less likely to have dental insurance or receive routine preventive dental care [15,16,17]. In Massachusetts, 1.7 million low-income residents are beneficiaries of MassHealth (which is what Medicaid is named in the state of Massachusetts), which provides comprehensive dental benefits including exams, cleanings, restorations, and dentures for almost one-quarter of Massachusetts residents [18]. MassHealth benefits also include nonemergency transportation services to dental providers for eligible recipients who are not able to access public transportation and/or private means of transportation.

Our study found that individuals with student provider-reported financial limitations were less likely to continue seeking dental care at a dental school. This finding is consistent with previous studies which suggest that cost is the most common reason why Americans do not seek dental care [19]. Americans unable to afford dental care are at higher risk of poor oral health outcomes such as tooth loss [20]. Although MassHealth’s coverage of comprehensive dental care has led to increased dental utilization, individuals without insurance or who are enrolled in Medicaid are still more likely to present to hospital emergency departments for urgent dental treatment [21,22]. 

The number of Americans with limited English proficiency (LEP) has increased by 80% since 1990, and 9% of the United States population now has LEP (25.2 million) [23]. LEP individuals tend to have lower income and poorer oral health [24]. Previous research has indicated that dental schools may see more LEP patients than the national average [25]. Interestingly, in this study, the proportion of patients with LEP seen in the teaching practice, 7.97%, was found to be lower than the proportion of Massachusetts residents with LEP, 8.8% [26]. This finding may be because the disposition forms asked students only to indicate patients whose treatment presented a “language barrier”, while the federal definition of LEP includes individuals who do not speak English “very well”. Additionally, awareness of dental school clinic services among LEP individuals may be lower, or enrollment to pursue care at a dental school clinic may be more difficult for people with LEP.

The most common languages spoken by LEP patients in the teaching practice, Spanish and Portuguese, represent the two most common languages spoken in Massachusetts other than English [26]. The third most commonly spoken language in Massachusetts is Mandarin Chinese. Spanish speakers compose 38.3% of Massachusetts’ LEP population and composed 43.05% of this study’s sample. Russian was the third most frequently spoken language among dental patients, though it is not one of the five most common languages spoken in Massachusetts. Vietnamese is currently the fourth most frequently spoken language in Massachusetts, but no Vietnamese speakers were present in this study set.

The most recent standards published by the Commission on Dental Accreditation (CODA) require dental school graduates to exhibit cultural competence, defined as “the ability to provide care to patients with diverse backgrounds, values, beliefs, and behaviors” [6]. Numerous didactic cultural competence curricula have been proposed for dental students [27,28,29]. However, dental students’ reflections indicate that it is through clinical interactions that the values innate to cultural competence are most often acquired [30]. Alarmingly, students’ attitudes towards poor and indigent populations tend to worsen as they progress through dental training and their clinical exposure increases [30].

The perceived respect for and acceptance of diversity by their dental school was previously found to be a predictor of dental students’ preparedness to treat diverse populations in the future [31]. Dental school faculty have acknowledged the importance of cultural competence; however, only 37.8% of those surveyed in a 2013 study indicated readiness to implement the CODA standard pertaining to cross-cultural communication [32]. If dental school clinics represent an environment less welcoming to those whose circumstances make access to care more difficult, they may be preventing optimal student preparedness to serve as culturally competent dentists. Since student experience working with vulnerable groups is a predictor of service to these groups in the future, dental school clinic experiences play a role in addressing future oral health disparities as well [7].

In our study, low-income and LEP patients elected to continue care less frequently. This suggests that changes in the current DSC environment or the process of obtaining care at a DSC may make dental care in this setting more accessible for these groups. The Harvard School of Dental Medicine has no formal interpreter services available for LEP patients, and this situation is consistent with the presence of less-than-ideal interpretation practices previously documented to occur in many DSCs in the US [25,33]. Patient satisfaction has been shown to be higher when professional interpreter services are made available, with satisfaction equally high when telephone interpreter services are used as when in-person interpreters are present [34]. One additional factor that the case disposition form does not consider is the effectiveness of culturally competent care provision. It may not be the patient’s language barrier, but instead the student providers’ lack of cultural competence, that leads to discontinuation of care. Exposure to interpreter services is also associated with increased provider comfort with their use, furthering student cultural competence [35]. Even though dental school clinic fees are lower than those found in private dental offices, these costs may still be prohibitively high for lower-income patients and patients with dental disease requiring complex and costly treatment plans. 

Satisfaction surveys used to evaluate patient care in the dental school setting have often been based on surveys previously validated for consumers in other dental settings [36]. Of note, measures of provider–patient interaction and its perceived quality are absent from these surveys, although patients drawn to the dental school setting may find these qualities more important than patients who seek care in a conventional dental setting [8]. While the attributes of dental school patients have been previously described, the studies have focused on specific groups, such as those with mental illness, substance abuse disorders, cancer, or hypertension [9,10,11,12]. Income and enrollment in insurance have previously been documented to predict the receipt of preventive dental services in the dental school setting; however, to our knowledge, this is the first study assessing the factors that lead dental school patients to depart from this care model [36].

On one hand, as it is increasingly recognized that patient outcomes are best when they have access to a permanent ‘dental home’, the majority of patients elected to continue care in the dental school setting after their established student provider graduated. On the other hand, it is concerning to note that the dental school clinic is least successful at establishing a dental home for patients at high risk of poor oral health, who may benefit most from this structure [37]. Moreover, the patients who declined continuing care in this study represent those patients who have already progressed through two required screenings and intake procedures, which themselves have high dropout rates [38]. Patients seeking care in the dental school setting may be attracted by lower prices and the clinic’s acceptance of public insurance. For example, all three dental schools in Massachusetts accept MassHealth, which is accepted by only 42% of dentists in the state [39]. Given the poorer oral health outcomes documented in these populations, dental schools should consider assisting patients in navigating the dental care system or piloting novel payment options to allow patients to complete necessary care and attain an optimal state of oral health [40].

However, it is important to note that this project was not originally designed to be a research study. The patient disposition form was developed as a quality improvement tool to transfer critical information from the current active provider to the new provider, which may enable a smoother transition for the patient. Since it is not primarily a research study, there are several limitations that must be considered when interpreting the results. Firstly, there was no age or gender information recorded because these factors were not critically important in smoothing the transition for patients. Secondly, the current study is only a preliminary survey consisting of data for the 2012 and 2013 graduating classes. Analysis including subsequent graduating classes may bear different results, especially as the dental services provided by MassHealth have been expanded in this time [14]. It is not known whether patients who elected not to continue care chose to pursue it in a different setting, or whether these patients did not seek further dental care at all. All data was self-reported by student providers who may have had different thresholds for what constituted constraints in obtaining care based on their own biases and experiences. Additionally, all barriers were recorded in a binary fashion. There may be several levels of severity for many of these barriers; for example, a patient unable to afford a dental implant would be recorded as having financial barriers identical to those faced by an indigent patient with no financial means for dental treatment. However, the significance of these variables in predicting continuing care indicates that even smaller challenges may make obtaining optimum care difficult for dental school patients.

## 5. Conclusions

Dental schools have a unique opportunity to provide valuable educational exposure to their students and serve as community partners in improving oral health and expanding access to care. Based on the results of this study, most dental school patients are satisfied with their care and choose to continue treatment in the dental school setting. However, patients at highest risk for poor oral health were those who were least likely to continue treatment when their student provider graduated. Dental schools should consider implementing interventions to assist these patients in obtaining treatment, such as patient navigators, payment plans, and interpreter services. Additionally, activity that facilitates better communication between the new provider, the patient, and the previous provider may aid in creating a smooth transition for the patient.

## Figures and Tables

**Table 1:** Summary of student-reported patient barriers to care.

Sample	Number (%)	Continuing Care	Not Continuing Care	*p* Value
Total sample size	1894	1391	503	
Patients with transportation barriers	118 (6.23%)	74 (5.3%)	44 (8.7%)	0.006 *
Patients with time limitations	290 (15.3%)	220 (15.8%)	70 (13.9%)	0.31
Patients with linguistic barriers	151 (7.97%)	85 (6.1%)	65 (12.9%)	<0.001 *
Patients with financial barriers	570 (30.1%)	348 (25%)	222 (44.1%)	<0.001 *

* Asterisk denotes statistically significant (*p* < 0.05).

**Table 2:** Languages spoken by patients with limited English proficiency.

Language	Number (%)
Total patients with limited English proficiency	151
Spanish	65 (43.05)
Portuguese	34 (22.52)
Russian	5 (3.31)
Mandarin	4 (2.65)
Haitian Kreyol	4 (2.65)
Hindi	2 (1.32)
Cantonese	2 (1.32)
Italian	2 (1.32)
Korean	2 (1.32)
Japanese	2 (1.32)
Romanian	2 (1.32)
Arabic	1 (0.66)
American Sign Language (ASL)	1 (0.66)

**Table 3:** Significance of barriers to continuing care.

Barrier	Significance of Association with Continued Care (Chi-Squared Test)	OR (95% CI)
Financial barrier	<0.001 *	0.454 (0.365–0.564) *
Time limitation	0.310	-
Transportation barrier	0.006 *	0.737 (0.493–1.10)
Linguistic barrier	<0.001 *	0.545 (0.384–0.774) *

* Asterisk denotes statistically significant (*p* < 0.05).

**Table 4:** Constraints distributed by chief complaint.

Chief Complaint	Financial Constraints	Time Constraints	Transportation Constraints	Language Barrier	Continuing Care?	Transfer Out of Predoc	Recall	Totals
(none)	317	161	60	76	845	47	447	**1953**
Broken Tooth	1	1	0	0	3	0	1	**6**
Bruxism	0	0	0	0	1	0	0	**1**
Crown	55	28	6	16	123	7	67	**302**
Dentures	41	11	17	15	92	6	45	**227**
Extraction	2	2	1	0	4	0	4	**13**
Filling	34	22	7	5	79	3	39	**189**
FPD	16	5	0	2	19	3	7	**52**
Hypertension, Diabetes	0	1	0	0	1	0	0	**2**
Implant	21	21	8	9	94	8	39	**200**
Missing Tooth	12	8	4	3	25	0	14	**66**
Pain	44	18	9	14	59	5	27	**176**
Periodontal Disease	5	1	1	1	8	0	6	**22**
Prophy	0	0	0	0	1	0	1	**2**
RCT	7	1	0	2	8	1	5	**24**
RPD	15	10	5	8	28	1	12	**79**
Sealants	0	0	0	0	1	0	1	**2**
Veneers	0	0	0	0	0	0	0	**0**
**Totals**	**570**	**290**	**118**	**151**	**1391**	**81**	**715**	**3316**

## Author Statement

The study protocol was reviewed and approved as “not human subjects research” by the Harvard School of Dental Medicine Institutional Review Board (IRB15-0341).
